# Optimization of human corneal endothelial cell culture: density dependency of successful cultures *in vitro*

**DOI:** 10.1186/1756-0500-6-176

**Published:** 2013-05-03

**Authors:** Gary SL Peh, Kah-Peng Toh, Heng-Pei Ang, Xin-Yi Seah, Benjamin L George, Jodhbir S Mehta

**Affiliations:** 1 Tissue Engineering and Stem Cell Group, Singapore Eye Research Institute, 11 Third Hospital Ave, #06-00, Singapore 168751, Singapore; 2 Singapore National Eye Centre, 11 Third Hospital Ave, #08-00, Singapore 168751, Singapore; 3 Department of Clinical Sciences, Duke-NUS Graduate Medical School, 8 College Road, Singapore, 169857, Singapore

**Keywords:** Human corneal endothelium, Human corneal endothelial cells, Primary cell culture, Cell density

## Abstract

**Background:**

Global shortage of donor corneas greatly restricts the numbers of corneal transplantations performed yearly. Limited *ex vivo* expansion of primary human corneal endothelial cells is possible, and a considerable clinical interest exists for development of tissue-engineered constructs using cultivated corneal endothelial cells. The objective of this study was to investigate the density-dependent growth of human corneal endothelial cells isolated from paired donor corneas and to elucidate an optimal seeding density for their extended expansion *in vitro* whilst maintaining their unique cellular morphology.

**Results:**

Established primary human corneal endothelial cells were propagated to the second passage (P2) before they were utilized for this study. Confluent P2 cells were dissociated and seeded at four seeding densities: 2,500 cells per cm^2^ (‘LOW’); 5,000 cells per cm^2^ (‘MID’); 10,000 cells per cm^2^ (‘HIGH’); and 20,000 cells per cm^2^ (‘HIGH^×2^’), and subsequently analyzed for their propensity to proliferate. They were also subjected to morphometric analyses comparing cell sizes, coefficient of variance, as well as cell circularity when each culture became confluent. At the two lower densities, proliferation rates were higher than cells seeded at higher densities, though not statistically significant. However, corneal endothelial cells seeded at lower densities were significantly larger in size, heterogeneous in shape and less circular (fibroblastic-like), and remained hypertrophic after one month in culture. Comparatively, cells seeded at higher densities were significantly homogeneous, compact and circular at confluence. Potentially, at an optimal seeding density of 10,000 cells per cm^2^, it is possible to obtain between 10 million to 25 million cells at the third passage. More importantly, these expanded human corneal endothelial cells retained their unique cellular morphology.

**Conclusions:**

Our results demonstrated a density dependency in the culture of primary human corneal endothelial cells. Sub-optimal seeding density results in a decrease in cell saturation density, as well as a loss in their proliferative potential. As such, we propose a seeding density of not less than 10,000 cells per cm^2^ for regular passage of primary human corneal endothelial cells.

## Background

The human cornea is a transparent dome-like disc found on the anterior segment of the eye, and is responsible for the refraction of light to the retina in the posterior eye for visual detection. This clear tissue consists of three cellular layers: the epithelium, stroma, and endothelium, and are separated by two acellular membranes (Bowman’s and Descemet’s) [1]. The role of the mono-layered corneal endothelium is to regulate corneal hydration, and dysfunction of this critical cellular layer will gradually result in corneal opacification and eventually results in loss of vision and corneal blindness [2-4]. Corneal transplantation is the only option available to restore vision. However, global shortage of available donor graft material and an ageing population requiring transplants, restricts the numbers of corneal transplants performed yearly [4]. This necessitates development of suitable graft alternatives through tissue engineering or a potential corneal endothelial cell replacement therapy through the injection of cultivated corneal endothelial cells (CECs) [5,6]. In order to facilitate the research and development of the above-mentioned studies, a robust approach that enables consistent propagation of isolated primary human corneal endothelial cells (HCECs) *in vitro*, to obtain sufficient numbers, is required.

Cells of the human corneal endothelial layer are not known to actively proliferate within the eye, and have been found to be arrested in the G1-phase of the cell cycle [7]. Contact-dependent inhibition, together with factors found within the aqueous humor, keep the corneal endothelium in a non-proliferative state [8,9]. However, *ex vivo* mechanical wounding studies and treatment of HCECs using EDTA to disrupt cell-to-cell contact have shown that these cells retain the capacity to proliferate [10,11]. The isolation and cultivation of HCECs *in vitro* have been reported by many groups, some with more apparent success than others [4]. Varying factors from isolation techniques, differing basal media, diverse range of supplements (including different types of growth factors and the concentration of bovine serum used), to individual donor cornea variability accounts for much of the mixed results [4]. In our previous study designed to negate potential donor cornea variability, we showed that the growth of CECs isolated from a single donor behaves differently when placed in culture medium of different formulations [12]. In that study, we identified two culture media, coded in that study as M2 [13] and M4 [14], to be able to support the active proliferation of isolated HCECs. Interestingly, some of the established primary HCEC-cultures showed differential growth preference for the two proliferative culture media. While most isolated HCECs grew relatively well in either of the medium, some samples displayed a marked preference for one medium over the other [12]. With such complexity involved, a systematic approach is required to be able to further improve the cultivation of HCECs *in vitro*. For example, it has been postulated that HCECs can be propagated on non-coated cell culture ware [15], but the use of culture ware pre-coated with extracellular matrices, such as a commercially available serum-free coating solution containing fibronectin, collagen and albumin (FNC coating mixture), greatly improved the attachment and subsequent expansion of the isolated HCECs [12,16]. More recently, it has been reported that the addition of a selective ROCK inhibitor Y-27632 enhanced cell adhesion and proliferation of CECs isolated from cynomolgus monkeys, which translated to improved cell survival and enhanced cell engraftment for CEC-based regenerative therapy [17,18].

Previously, it has been reported that the growth pattern of CECs isolated from macaque monkeys is affected by initial cell seeding density, suggesting that successful CEC-culture may be density dependent [19]. To our knowledge, the density dependent growth of HCECs and its effect on *in vitro* expansion has not been described. The aim of this study was to investigate the density dependency of the growth of primary HCECs isolated from pairs of donor corneas and its implication for a robust cell expansion strategy in order to obtain sufficient numbers of *bona fide* primary cells for downstream development of a tissue-engineered graft alternative or cell injection therapy.

## Methods

### Materials

Ham’s F12, Medium 199, Human Endothelial-SFM, fetal bovine serum (FBS), Dulbecco’s Phosphate-Buffered Saline (PBS), TrypLE Express (TE), 100× anti-biotic/anti-mycotic solution were purchased from Invitrogen (Carlsbad, CA, USA). Insulin, transferrin, selenium (ITS), ascorbic acid, trypan blue (0.4%) were purchased from Sigma (St. Louis, MO, USA). FNC coating mix was purchased from United States Biologicals (Swampscott, MA, USA). Collagenase A was obtained from Roche (Mannhein, Germany).

### Ethics statement

The following protocols conformed to the tenets of the Declaration of Helsinki, and written consent was acquired from the next of kin of all deceased donors regarding eye donation for research. This study was approved by the institutional review board of the Singapore Eye Research Institute/Singapore National Eye Centre.

### Research-grade human corneoscleral tissues

Three pairs of research-grade cadaver human corneas were procured from Lions Eye Institute for Transplant and Research Inc. (Tampa, FL, USA) and preserved in Optisol-GS at 4°C. All corneas used in this study had an endothelial cell density count of over 2500 cells per mm^2^ and were processed within 10 days of preservation. Donor ages were 19, 31 and 35 years old (Table 1).

**Table 1:** Donor information

**Serial number**	**Age**	**Sex**	**Days to culture**	**Cell count (OS/OD)**	**COD**
01	19	F	7	2681/2882	Acute Cardiac Crisis
02	31	F	9	2591/2611	Overdose
03	35	F	5	2899/2941	Overdose

Number days taken from death of donor to the initiation of corneal endothelial cell culture. COD: cause of death. OS: oculus sinister. OD: oculus dexter.

### Isolation and growth of human corneal endothelial cells

Primary cultures were isolated from human corneoscleral tissues as described previously [12] with some modifications in the way the isolated HCECs were cultured for expansion. Briefly, corneas were washed three times in a 1× anti-biotic/anti-mycotic solution in PBS (wash buffer) for 15 minutes. Cells of the corneal endothelium were isolated using a two-step “peel-and-digest” approach. A disposable vacuum donor punch (Ripon, England) was used to hold the corneoscleral rims in place, endothelial cell-side up. A short 30 seconds treatment with 0.1% trypan blue solution (diluted in PBS), on the corneal endothelial cell surface was used to outline the Schwalbe’s line. Using sterile surgical forceps, the sheet of Descemet’s membrane with intact endothelium, approximately 0.5 to 1mm posterior to the Schwalbe’s line was carefully removed and incubated in collagenase A (2 mg/ml) at 37°C for at least 4 hours (up to 6 hours) to dislodge the corneal endothelial cells from the Descemet’s membrane. Dislodged corneal endothelial cell-clusters were rinsed once in PBS and further dissociated with a brief treatment of TE for 5 minutes to obtain smaller cell-clumps. The cell clumps were washed and collected after centrifugation at 0.8 *g* for 5 minutes and plated on FNC-coated tissue culture dishes for attachment. Isolated cells were left to adhere overnight in a stabilization medium made up of Human Endothelial-SFM supplemented with 5% FBS and 1× anti-biotic/anti-mycotic. Adhered HCECs were then cultured in F99 medium containing Ham’s F12 and M199, mixed in a 1:1 ratio, supplemented with 5% FBS, 20 μg/ml ascorbic acid, 1× ITS, 1× anti-biotic/anti-mycotic and 10 ng/ml bFGF. When the cultured cells reached 80-90% confluence, they were exposed to the stabilization medium for at least one week before passage. The inclusion of this step enhanced the morphology of the expanded HCECs (unpublished observation; manuscript in preparation). Cultured HCECs were passaged using TE, and sub-cultured at a seeding density of 10,000 cells per cm^2^ for each passage and were used at the third passage for this study. At the second passage, cultured HCECs were dissociated and plated at the following seeding densities: 2,500 cells per cm^2^ (‘LOW’), 5,000 cells per cm^2^ (‘MID’), 10,000 cells per cm^2^ (‘HIGH’), and 20,000 cells per cm^2^ (‘HIGH^×2^’). Cells were then cultured for at least 10 days before morphometric analysis. All incubation and cultures of HCECs were carried out in a humidified incubator at 37°C with 5% CO_2_ and fresh medium was replenished every two days.

### Immunocytochemistry and antibodies

Confluent cultures of primary HCECs grown on glass coverslips at the second passage were fixed in 100% ice-cold ethanol for 5 minutes. The staining procedure involved immersion of the fixed sample in a block solution of PBS containing 10% normal goat serum for 30 minutes. Samples were subsequently incubated with the primary antibody for an hour, followed by a secondary antibody in the dark for 30 minutes at room temperature. Between incubations, samples were rinse twice within PBS. Labeled samples were mounted onto glass slides in Vectashield containing DAPI (Vector Laboratories, Burlingame, CA, USA) to counter-stain cell nuclei. Fluorescence images were captured using a Zeiss Axioplan 2 fluorescence microscope (Carl Zeiss, Germany). The primary antibodies used in this study were: mouse IgG_1_ anti-Na^+^K^+^/ATPase α1 (5 μg/mL; Santa Cruz Biotechnology) and mouse IgG_1_ anti-ZO-1 (5 μg/mL; BD Biosciences Pharmingen). Secondary antibody used was Alexa Fluor 488 goat anti-mouse IgG (2 μg/mL; Life Technologies). Negative controls were cells incubated with an anti-mouse IgG_1_ isotype control (5 μg/mL; BioLegends) in place of the primary antibody.

### Morphometric analysis and time-lapse imaging

Cellular morphology of cultured HCECs was captured using a Nikon TS1000 phase contrast microscope with a Nikon DS-Fil digital camera (Nikon, Japan). Morphometric data of the area and perimeter of randomly selected cells from phase contrast images of each seeding density was manually outlined by point-to-point tracing of the cell borders using ImageJ software [20]. Cell circularity was then determined using the formula: $\text{Circularity}=\frac{4\pi \times \text{Area}}{\text{Perimete}r^{2}}$, where a value approaching 1.0 indicates a circular profile. Hence, hexagonal HCECs will have a profile closer to 1.0 compared to long and spindly fibroblast-like HCECs. At least 100 HCECs from each condition (*n* = 3) were analyzed. For time-lapse imaging, HCECs were seeded onto FNC-coated 35 mm dishes and transferred into a time-lapse imaging system: Biostation IM-Q (Nikon, Japan). The incubator chamber within was maintained at 37°C and 5% CO_2_. Viewing area was selected manually and the system was setup to take images automatically every 30 minutes for 24 hours under both 10× and 20× objective lenses. Images were exported from the Biostation IM-Q format and compiled into video using Avidemux software (http://fixounet.free.fr/avidemux/).

### Cell proliferation assay

The proliferation of HCECs grown at 4 different seeding densities at the third passage was assessed using Click-iT™ EdU Alexa Fluor 488 Imaging kit (Invitrogen). This assay measures the incorporation of EdU (5-ethynyl-2’-deoxyuridine) into DNA during active DNA synthesis. Cultured HCECs were sub-cultured onto FNC-coated glass slides at the four seeding densities of 2,500 cells per cm^2^, 5,000 cells per cm^2^, 10,000 cells per cm^2^, and 20,000 cells per cm^2^ overnight to allow cell attachment. Adhered HCECs were then treated with 10 uM EdU solution for 24 hours. After treatment, cells were fixed in 4% paraformaldehyde (PFA) for 15 minutes at room temperature, rinsed twice with 3% BSA in PBS, and permeabilized with 0.1% Triton X-100 in PBS for 20 minutes at room temperature. Click-iT™ reaction cocktail used to detect the incorporated EdU was made by combining 1× Click-iT™, CuSO_4_, Alexa Fluor azide and the reaction buffer additive provided in the kit. Samples were incubated in the reaction cocktail for 30 minutes at room temperature in the dark. After two rinses with 3% BSA in PBS, samples were mounted on glass slides with Vectashield containing DAPI. Fluorescence images were captured using a Zeiss Axioplan 2 fluorescence microscope. At least 100 nuclei were analyzed randomly for each donor set (*n* = 3).

### Statistics

All numeric data obtained are expressed as mean ± standard deviation. Comparisons of HCECs sizes, cell circularity and cell proliferation were statistically analyzed using two-way ANOVA followed by post-hoc Bonferroni test for multiple comparisons (SPSS Statistics 17.0, IBM, Chicago, IL, USA). Comparison of Day 10 and Day 30 sizes of HCECs were performed using an independent sample t-test. Results with a *p*-value of less than 0.05 were deemed to be statistically significant.

## Results

### Isolation and cultivation of primary HCECs

**Figure 1:**
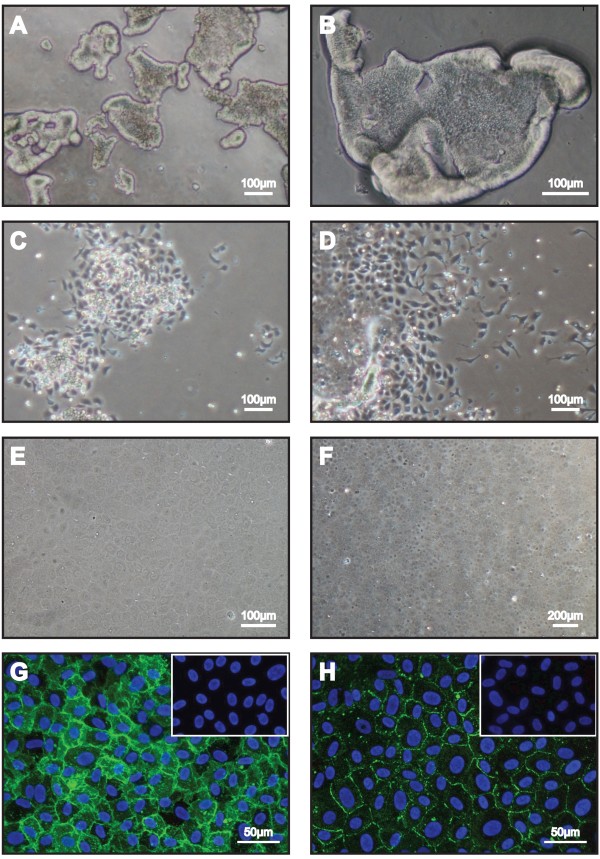
**Isolation, cultivation and characterization of primary HCECs. A**) Compact clusters of HCECs were released from the DM following approximately 5 hours of collagenase (2 mg/ml) treatment. **B**) A high magnification micrograph of dislodged HCECs sheet rounding up at the edges. **C**) Attachment of HCEC onto FNC-coated dish in a dispersed-clusters manner at Day 1 in the stabilization medium. **D**) Outspread and migration of HCECs in the proliferative medium from the second medium change onwards. **E **&**F**) Confluent HCECs displayed distinct cell borders and uniform cell shape in stabilization medium at Day 14. Confluent cultures of HCECs at the second passage express cellular markers indicative of the human corneal endothelium: **G**) Na^+^K^+^ATPase and native isotype-matched control (insert); as well as **H**) ZO-1 together with its native isotype-matched control (insert).

The isolation of HCECs from human donor cadaver research-grade corneas were achieved using a two-step ‘peel and digest’ approach as previously described [12]. Peeled Descemet’s membrane (DM), together with the corneal endothelium was exposed to collagenase for at least 4 hours and up to 6 hours, to dislodge the cells of the corneal endothelium from the DM, which in turn aggregated into HCEC-clusters of various sizes (Figure 1A and 1B). Further treatment with TrypLE Express (TE) dissociated the larger cell clusters into smaller cell clumps and single cells. Isolated cells from each pair of donor corneas were plated onto one FNC-coated well of a 6-well plate (with a growth area of approximately 9.6 cm^2^), and allowed to adhere in a stabilization medium for 24 hours (Figure 1C). Upon attachment, the established HCECs were cultured in F99 medium to promote the proliferation of HCECs Within the next 24 to 36 hours, extensive proliferation of HCECs migrating out from the initial site of attachment was observed (Figure 1D). Once the proliferating HCECs became 80% to 90% confluent, the cells were re-introduced to the stabilization medium for at least 1 week, which enabled the HCECs to retain their corneal endothelium-like cellular morphology (Figure 1E and 1F). Primary cultures from all three donors were then sub-cultured using TE to dissociate the cells, and re-seeded at a plating density of approximately 10,000 cells per cm^2^ from P0 to P1, and subsequently, from P1 to P2 using this approach. We were able to achieved consistent culture of P2 cells displaying distinct cellular borders and uniform polygonal/hexagonal cellular morphology. These cells expressed classical cellular markers indicative of the human corneal endothelium: sodium potassium pump - Na^+^K^+^ATPase (Figure 1G), and tight junctional protein - ZO1 (Figure 1H).

### Morphometric assessment of P3 HCECs (Day 10) cultured at four plating densities

**Figure 2:**
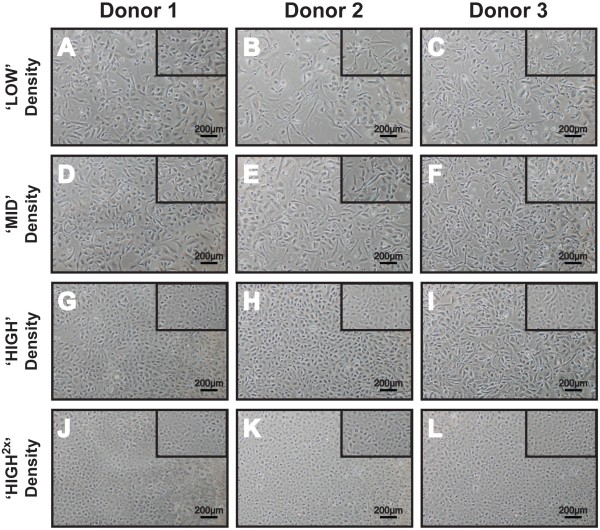
**Morphology of HCECs plated at the four seeding densities. **Dissociated P3 HCECs from all three donors were plated at four different densities: ‘LOW’ (2,500 cells per cm^2^), ‘MID’ (5,000 cells per cm^2^), ‘HIGH’ (10,000 cells per cm^2^) and ‘HIGH^2×^’ (20,000 cells per cm^2^). Morphological images of cultured HCECs were captured at Day 5 and Day 10 (insert).

**Figure 3:**
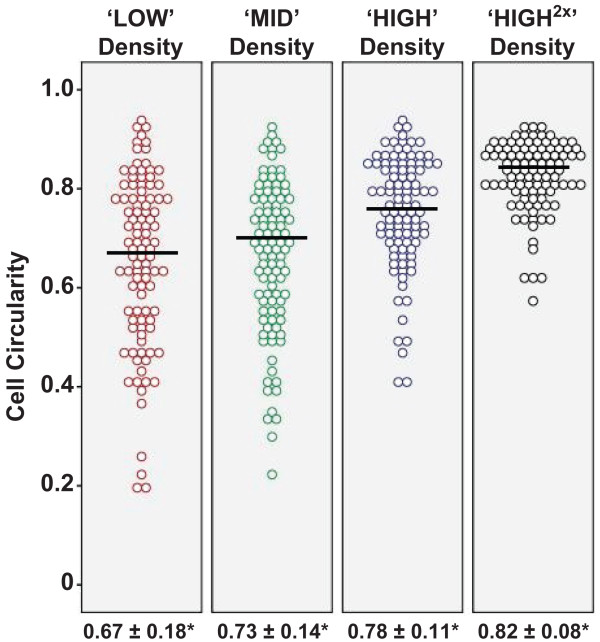
**Cellular circularity of HCECs plated at the four seeding densities. **Determination of cellular circularity of HCECs cultured at the four plating densities were carried out at Day 10. Generally, cellular circularity showed rounder cells with increased plating density, indicative of a preservation of hexagonal cellular morphology at higher seeding densities. Statistical comparisons were performed using two-way ANOVA followed by post-hoc Bonferroni test for multiple comparisons, and significance were observed between all groups ^*^p < 0.01.

The cellular morphology of cultured P3 HCECs was examined using phase contrast microscopy where three donors, each with four seeding densities were examined at Day 5 following attachment. Insert within each figure shows representative HCECs at Day 10 cultured at their respective seeding densities (Figure 2), where the morphometric data for analyses were collected (Table 2). At Day 5 and Day 10, cultures of HCECs that were seeded at lower densities (Figure 2A-F) were less confluent than HCECs cultured at higher densities (Figure 2G-L). This observation is consistent across all three donors. Morphologically, HCECs seeded at the ‘LOW’ density remained dispersed, were the largest (8117.83 ± 4396.84 μm^2^) and displayed heterogeneous cells with high coefficient of variance (CV: 0.54) that were more elongated with a cell circularity index of 0.67 ± 0.18 at Day 10 (Figure 3). Cultured P3 HCECs plated at the ‘MID’ density were significantly smaller in size (5997.57 ± 2571.47 μm^2^) and less variable (CV: 0.43) than those seeded at the ‘LOW’ density. Although cells from the ‘MID’ seeding density had a higher circularity index of 0.73 ± 0.14 (Figure 3), heterogeneous cellular morphology was still seen.

**Table 2:** Cell size and CV of cultured P3 HCECs at Day 10

**Seeding density**	**Cell size ± SD (μm**^**2**^**)**	**Coefficient of variation (CV)**
‘LOW’ Density	8117.83 ± 4396.84^*^	0.54
‘MID’ Density	5997.57 ± 2571.47^*^	0.43
‘HIGH’ Density	5010.97 ± 2003.53^*^	0.40
‘HIGH^2×^’ Density	3440.30 ± 1236.58^*^	0.36

The cell sizes and coefficient of variation of P3 HCECs initiated at the four seeding densities were taken at Day 10. Statistical comparisons were performed using two-way ANOVA followed by post-hoc Bonferroni test for multiple comparisons, and significance were observed between all groups ^*^p < 0.01.

When cultured HCECs from the same series of donors were plated at the two higher seeding densities, at Day 10, they were found to be smaller in size, with a relatively homogenous compact cellular morphology. Specifically, HCECs seeded at the ‘HIGH’ density had an average cellular size of 5010.97 ± 2003.53 μm^2^, a CV of 0.40 and a cell circular index of 0.78 ± 0.11 (Figure 3). HCECs seeded at the highest plating density (‘HIGH^2×^’) were distinctly the most compact (3440.30 ± 1236.58 μm^2^), and were the most homogeneous (CV: 0.36) and hexagonal in shape as suggested by their cellular circularity index of 0.82 ± 0.08 (Figure 3).

### Morphometric assessment of P3 HCECs (Day 30) cultured at lower plating densities

The primary HCECs that were passaged at the two lower seeding densities were cultured up to Day 30 and re-analyzed. Comparatively, measurement taken at Day 30 showed that HCECs became significantly larger, from 8117.83 ± 4396.84 μm^2^ to 9470.16 ± 3825.78 μm^2^ (‘LOW’) and 5997.57 ± 2571.47 μm^2^ to 8299.53 ± 3408.87 μm^2^ (‘MID’) suggesting that there was a lack of proliferation, and these cells were unable to form a compact monolayer as seen in cells plated at the two high densities, suggesting that HCECs cultured at both ‘LOW’ and ‘MID’ seeding densities may not be optimal to ensure the continual expansion of cultured HCECs with uniform and polygonal-shape cell morphology. Interestingly, both CV and cell circularity values improved somewhat suggesting the occurrence of cell structure rearrangement, where cells became less variable and rounder (Table 3).

**Table 3:** Cell size, CV and cellular circularity of cultured P3 HCECs at Day 30

**Seeding density**	**Cell size ± SD (μm**^**2**^**)**	**Coefficient of variation (CV)**	**Cellular circularity**
‘LOW’ Density	9470.16 ± 3825.78^*^	0.40	0.79 ± 0.12
‘MID’ Density	8299.53 ± 3408.87^*^	0.41	0.78 ± 0.08

The average cell sizes, CV and cellular circularity of P3 HCECs seeded at the two lower densities, analyzed at Day 30. Statistical comparisons were performed using independent sample t-tests, and significance were observed between the groups compared ^*^p < 0.01.

### Cell proliferation assay

**Figure 4:**
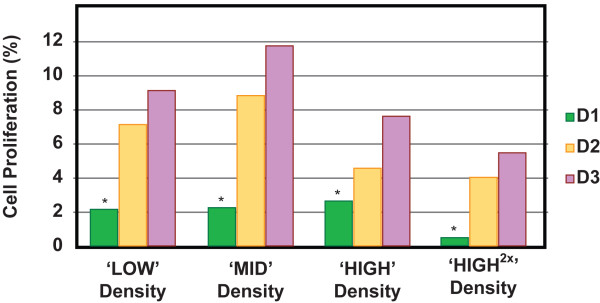
**Percentages of proliferative P3 HCECs in the four seeding densities. **HCECs were seeded at the densities of ‘LOW’, ‘MID’, ‘HIGH’, and ‘HIGH^2×^’. Proliferation rates of HCECs were assessed using Click-iT assay. D1: Donor 1. D2: Donor 2. D3: Donor 3. The differences in proliferation rates were not statistically significant. However, ANOVA analysis showed significant donor-to-donor variation (*p* < 0.05).

The percentages of proliferative HCECs seeded at 4 different densities were assessed using Click-iT™ EdU assay. As a significant donor-to-donor variation was observed, for D1 (Donor 1), with cell proliferation rates of lesser than 2.6% for all seeding densities, this data set was presented as individual donor sample sets: D1, D2, and D3 (Figure 4). Cells seeded at the ‘MID’ density were the most proliferative across the other two donors (D2: 8.8%; and D3: 11.7%), when compared to HCECs seeded at the ‘LOW’ density (D2: 7.1%; and D3: 9.1%) and ‘HIGH’ density (D2: 4.5%; and D3: 7.6%). HCECs seeded at the highest density were found to be the least proliferative (D2: 4.0%; D3: 5.5%). A possible explanation for the lower proliferation rates observed in the two higher densities could be due to the higher numbers of cells that were seeded, and that cell-to-cell contact was established faster, which in turn inhibited cell proliferation. Conversely, video time-lapse movie of HCECs behavior at low density showed extensive, but random cellular movement for the initial 24 hours, in an apparent attempt to establish proper cellular contact with adjacent cells, without much migration or expansion seen in HCECs cultured at higher density (Additional file 1: Movie S1). As the Click-iT EDU assay was initiated 24 hours after plated cells attached, and followed for another 24 hours, this may have accounted for the lower proliferation rate recorded in HCECs seeded at the ‘LOW’ density. Nevertheless, the proliferation rates reported were not statistically significant, and this may be due in part to the lower proliferation profile seen in Donor 1 across all four seeding densities, and in part to the low sample size.

### Projected cell numbers of cultured HCECs up to the third passage

The total number of cells obtained from each donor from the oculus dexter and ocular sinister were combined, and seeded to establish primary HCECs for propagation. Based on the central corneal endothelial cell density of the donor corneas (Table 1; average of 2768 cells per mm^2^), the total area of DM isolated (approximately 11mm width or an area of approximately 95.03 mm^2^), and accounting for approximately 10% cell death and another 10% cell loss, we estimated that the initial isolated numbers of HCECs to be approximately 4.25 × 10^5^ cells per pair of donor corneas. It should be noted that this estimation is highly dependent on the initial count of the central corneal endothelial cell density of the donor corneas obtained, and may be significantly affected by the final yield following donor sample preparation and the isolation process. Retrospectively, for this study, we were able to obtain approximately 8.0 × 10^5^ to 1.0 × 10^6^ cells at the end of P0. Based on the cell counts performed following cell dissociation, the cell suspension obtained were split at a ratio of approximately 1:3 to 1:4 so as to ensure a seeding density of at least 1.0 × 10^4^ cells per cm^2^. In this study, based on the average cell counts at each passage, including both the lowest and the highest numbers obtained from each donor, we projected to be able to obtain between 1.0 × 10^7^ to 2.5 × 10^7^ cultured HCECs at the end of the third passage (Table 4). However, it should be noted that these projected values were based on the cell expansion numbers obtained from the three pairs of cornea used in this study, which varied significantly between donors.

**Table 4:** Projection of the range of HCECs obtainable each passage at confluent

**Passage no.**	**Projected HCECs at each passage**
P0	8.0 × 10^5 ^to 1.0 × 10^6^ cells
P1	2.1 × 10^6^ to 3.2 × 10^6^ cells
P2	4.5 × 10^6 ^to 7.5 × 10^6^ cells
P3	1.0 × 10^7 ^to 2.5 × 10^7^ cells

Based on the isolation protocol, the cultivation methodology described in this paper, and a seeding density of not less than 1.0 × 10^4 ^cells per cm^2^, it is possible to scale up the number of HCECs to a range between 1.0 × 10^7 ^to 2.5 × 10^7 ^cells at confluence, by the third passage from a pair of donor corneas.

## Discussion

The vision of patients affected by debilitating corneal blindness as a result corneal endothelial dysfunction can be restored by the replacement of the diseased or damaged corneal endothelium with a healthy donor cornea tissue through a corneal transplant. However, there is a global shortage of donor corneas available for transplants and many more are rejected due to low endothelial cell count, as well as potential cultural, logistical and technical difficulties [4,21]. To overcome the shortage of donor corneas, development of potential graft alternatives through a tissue bioengineering approach is currently of great clinical interest. However, the ability to consistently cultivate sizable numbers of HCECs *in vitro* is critical in stimulating further research in the development of such a bioengineered graft replacements.

Whilst a consensus has yet been established for the culture of HCECs, studies contributing to the improvements of their cultivation are ongoing. For example, recent studies using CECs isolated from non-human primates (cynomolgus monkey), conducted to investigate the applicability of Rho kinase inhibitor Y-27632 in promoting the cultivation of primate CECs, showed that Y-27632, at a concentration of 10 μM, promoted adhesion, inhibited apoptosis and increased the proliferation of these primate CECs [17]. The authors have since postulated the use of Y-27632 together with a “cell-injection therapy”, as a potential new therapy for patients with dysfunction of the corneal endothelium [5]. In a more recent study, Okumura and colleagues were able to reverse corneal opacification by an injection of 2 × 10^5^ cultivated rabbit CECs or 2 × 10^5^ cultivated monkey CECs into the anterior chambers of respective rabbit or monkey models of corneal endothelial dysfunction [18]. This translates to a seeding density of approximately 3,150 cells per mm^2^ within a circular area with a 9 mm diameter. As projected in this current study, using the culture strategy described, HCECs isolated from a pair of donor cornea can be expanded to between 4.5 × 10^6^ to 7.5 × 10^6^ cells at confluence by the second passage. Hypothetically, adopting the cell numbers used in the cell injection therapy (2 × 10^5^ cells per eye) reported by Okumura and colleagues [18], cultivated confluent human CECs obtainable at the second passage can potentially treat 22 to 37 cases of corneal endothelial dysfunction via cell-injection therapy. Alternatively, similar numbers of tissue-engineered HCEC-constructs can be potentially generated on either synthetic or biological carriers (reviewed in [4]) as alternative graft materials.

To improve the growth of CECs, it was reported in an earlier study that there is a significant relationship between cell density and the growth of primate CECs isolated from non-human primate (macaque monkey) [19]. To our knowledge, there is no published data showing cell density dependent growth for extended cultivation of primary HCECs. In this present study, the growth dynamics of cultivated HCECs was examined when expanded HCECs, from each donor at the second passage were plated out at 4 seeding densities in an attempt to delineate an optimal seeding density for their continual *in vitro* expansion. Based on cellular morphology, our results showed that there is a density dependency in the growth of primary HCECs. Lower seeding densities tend to encourage greater cell proliferation for the first few days, although this observation was not significant. As assessed by cell morphometric measurements at Day 10 in culture, HCECs seeded at lower densities were significantly larger in size, became heterogeneously variable in terms of their cellular shape, and contained mixtures of hexagonal HCECs, as well as enlarged or elongated fibroblast-like cells (Figure 2). Comparatively, HCECs from the same series of donors that were passaged at higher plating densities retained relatively compact cellular morphology, characteristic of the naïve corneal endothelium. This result is consistent with the findings reported for primate CEC-cultures [19]. Interestingly, HCECs plated at the low or medium densities were unable to form a compact monolayer even after extended culture for 1 month. Some form of cellular reorganization occurred as the cultures became more homogeneous and rounder when analyzed at Day 30. Such cellular reorganization and cellular spreading phenomena have has been reported *in vivo* where existing cells of the corneal endothelium spread out to maintain the functional integrity of the corneal endothelial layer to sustain corneal deturgescence and maintain corneal transparency as a way to replace dead or damaged CECs [22,23]. However, HCECs seeded at lower densities remained significantly larger compared to cells plated at higher densities (Table 3). This result can be inferred as an overall loss of proliferative potential [24]. The decrease in saturation density, together with an increase in cell size, as well as the loss of further division capability are also hallmarks of cellular senescence (review in [25]). However, it should be noted that cultivated HCECs are mediated in part by contact-induced inhibition [8]. Hence it is unclear if the loss of proliferative potential is due to premature cellular senescence or contact inhibition. Hence further studies to delineate the mechanisms that may be in play should focus on the gene signatures, protein expression or enzyme activity such as senescence-associated beta galactosidase, as well as the activity of p27kip1 in cultured HCECs that are plated at a lower seeding density.

## Conclusions

Our results demonstrated that the successful outcome of extended culture of primary HCECs is negatively impacted by lower, sub-optimal plating density, and can significantly affect their proliferative potential. Even though HCECs may be viable when seeded at lower densities, the quality of those cells was not comparable to cells that were sub-cultured at higher densities. From a pair of donor corneas, using the isolation methodologies and culture approach for the propagation of isolated primary HCECs described in this study, and following a seeding density of not less than 1 × 10^4^ cells per cm^2^, it is possible to obtain up to 2.5 × 10^7^ cells with preserved polygonal/hexagonal cellular morphology that resembled cells of the corneal endothelium at the end of the third passage. Whether cultivated HCECs should be utilized at the second or third passage is the subject of further functional characterization using both *in vitro* (cellular physiology) and *in vivo* (corneal dysfunction animal model) approach. Nevertheless, a robust culture strategy that can consistently produce a sizeable number cultivated *bone fide* primary HCECs is essential to facilitate the validation of cell-injection therapy, or downstream development of an alternative corneal endothelium construct through cell-tissue engineering.

## Competing interests

Singapore Patent Application No. 201205413-6 – The culture of Human Corneal Endothelial Cells using a Dual Media approach. The patent is not expected to provide any financial gain to the authors based on the publication of this manuscript.

## Authors’ contributions

KPT isolated and cultivated the primary HCECs, carried out the cell proliferation assay, coordinated the study and wrote the first draft. HPA and XYS participated in morphometric and statistical analysis. BLG performed the time-lapse experiments. GSP carried out the immunocytochemistry, and together with JSM conceived the study, designed the experiments and edited the draft. All authors read and approved the final manuscript.

## Supplementary Material

Additional file 1: Movie S1 Time-lapse images of HCECs from the same donor (P2) seeded at a high density (left) and at a low density (right), taken at an interval of 30 minutes for 24 hours. Images from the two densities were complied and stitched together using Avidemux software into a movie. Click here for file

## References

[B1] Klyce SD Beuerman RW Kaufman HE, Barron BA, McDonald MB, Waltman SR Structure and function of the cornea The Cornea 1988 New York: Churchill Livingstone 3 28

[B2] Laing RA Sanstrom MM Berrospi AR Leibowitz HM Changes in the corneal endothelium as a function of age Exp Eye Res 1976 22 6 587 594 10.1016/0014-4835(76)90003-8 776638

[B3] Geroski DH Matsuda M Yee RW Edelhauser HF Pump function of the human corneal endothelium. Effects of age and cornea guttata Ophthalmology 1985 92 6 759 763 2412197 10.1016/s0161-6420(85)33973-8

[B4] Peh GS Beuerman RW Colman A Tan DT Mehta JS Human corneal endothelial cell expansion for corneal endothelium transplantation: an overview Transplantation 2011 91 8 811 819 10.1097/TP.0b013e3182111f01 21358368

[B5] Koizumi N Okumura N Kinoshita S Development of new therapeutic modalities for corneal endothelial disease focused on the proliferation of corneal endothelial cells using animal models Exp Eye Res 2012 95 1 60 67 10.1016/j.exer.2011.10.014 22067130

[B6] Choi JS Williams JK Greven M Walter KA Laber PW Khang G Soker S Bioengineering endothelialized neo-corneas using donor-derived corneal endothelial cells and decellularized corneal stroma Biomaterials 2010 31 26 6738 6745 10.1016/j.biomaterials.2010.05.020 20541797

[B7] Joyce NC Meklir B Joyce SJ Zieske JD Cell cycle protein expression and proliferative status in human corneal cells Invest Ophthalmol Vis Sci 1996 37 4 645 655 8595965

[B8] Joyce NC Harris DL Mello DM Mechanisms of mitotic inhibition in corneal endothelium: contact inhibition and TGF-beta2 Invest Ophthalmol Vis Sci 2002 43 7 2152 2159 12091410

[B9] Joyce NC Proliferative capacity of corneal endothelial cells Exp Eye Res 2012 95 1 16 23 10.1016/j.exer.2011.08.014 21906590 PMC3261346

[B10] Senoo T Obara Y Joyce NC EDTA: a promoter of proliferation in human corneal endothelium Invest Ophthalmol Vis Sci 2000 41 10 2930 2935 10967047

[B11] Senoo T Joyce NC Cell cycle kinetics in corneal endothelium from old and young donors Invest Ophthalmol Vis Sci 2000 41 3 660 667 10711678

[B12] Peh GS Toh KP Wu FY Tan DT Mehta JS Cultivation of human corneal endothelial cells isolated from paired donor corneas PLoS One 2011 6 12 e28310 10.1371/journal.pone.0028310 22194824 PMC3241625

[B13] Zhu C Joyce NC Proliferative response of corneal endothelial cells from young and older donors Invest Ophthalmol Vis Sci 2004 45 6 1743 1751 10.1167/iovs.03-0814 15161835

[B14] Engelmann K Friedl P Growth of human corneal endothelial cells in a serum-reduced medium Cornea 1995 14 1 62 70 7712739

[B15] Chen KH Azar D Joyce NC Transplantation of adult human corneal endothelium ex vivo: a morphologic study Cornea 2001 20 7 731 737 10.1097/00003226-200110000-00012 11588426

[B16] Engler C Kelliher C Speck CL Jun AS Assessment of attachment factors for primary cultured human corneal endothelial cells Cornea 2009 28 9 1050 1054 10.1097/ICO.0b013e3181a165a3 19724204

[B17] Okumura N Ueno M Koizumi N Sakamoto Y Hirata K Hamuro J Kinoshita S Enhancement on primate corneal endothelial cell survival in vitro by a ROCK inhibitor Invest Ophthalmol Vis Sci 2009 50 8 3680 3687 10.1167/iovs.08-2634 19387080

[B18] Okumura N Koizumi N Ueno M Sakamoto Y Takahashi H Tsuchiya H Hamuro J Kinoshita S ROCK inhibitor converts corneal endothelial cells into a phenotype capable of regenerating in vivo endothelial tissue Am J Pathol 2012 181 1 268 277 10.1016/j.ajpath.2012.03.033 22704232

[B19] Arita T Okamura R Kodama R Takeuchi T Kadoya Y Eguchi G Density dependent growth of corneal endothelial cells cultured in vitro Cell Differ 1987 22 1 61 69 10.1016/0045-6039(87)90413-1 3690674

[B20] Schneider CA Rasband WS Eliceiri KW NIH Image to ImageJ: 25 years of image analysis Nat Methods 2012 9 7 671 675 10.1038/nmeth.2089 22930834 PMC5554542

[B21] Ruberti JW Zieske JD Prelude to corneal tissue engineering - gaining control of collagen organization Prog Retin Eye Res 2008 27 5 549 577 10.1016/j.preteyeres.2008.08.001 18775789 PMC3712123

[B22] Kaufman HE Katz JI Pathology of the corneal endothelium Invest Ophthalmol Vis Sci 1977 16 4 265 268 844984

[B23] Edelhauser HF The resiliency of the corneal endothelium to refractive and intraocular surgery Cornea 2000 19 3 263 273 10.1097/00003226-200005000-00002 10832681

[B24] Demidenko ZN Blagosklonny MV Quantifying pharmacologic suppression of cellular senescence: prevention of cellular hypertrophy versus preservation of proliferative potential Aging (Albany NY) 2009 1 12 1008 1016 20157583 10.18632/aging.100115 PMC2815749

[B25] Stanulis-Praeger BM Cellular senescence revisited: a review Mech Ageing Dev 1987 38 1 1 48 10.1016/0047-6374(87)90109-6 2439851

